# Supplementary material for: Utilizing Amino Acid Composition and Entropy of Potential Open Reading Frames to Identify Protein-Coding Genes
Source: Microorganisms. 2021 Jan 8;9(1):129. doi: 10.3390/microorganisms9010129 (PMC7827183; doi:10.3390/microorganisms9010129)
Supplement: Supplementary file 1 [file microorganisms-09-00129-s001.pdf]

Supplementary Figures

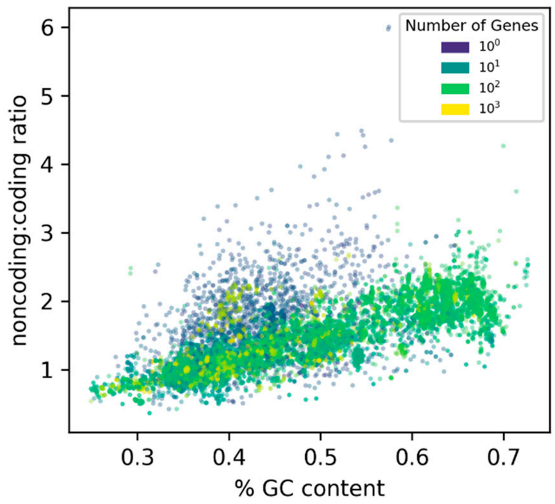

Supplementary Figure 1 Showing the relationship between GC content and the ratio of noncoding ORFs to coding. As the GC content increases, the chance of randomly encountering a stop codon (which are mainly T and A nucleotides) decreases. Thus there are more spurious noncoding ORFs. The ratio is found by taking all potential start codon trucations of noncoding ORFs and dividing by all the potential start codon locations of coding ORFs. The points are colored according to how many protein-coding genes the annotated genome has, showing that there are many small genomes (around 1-10 genes long) that do not follow the clear linear trend, and have many more noncoding ORFs than expected.

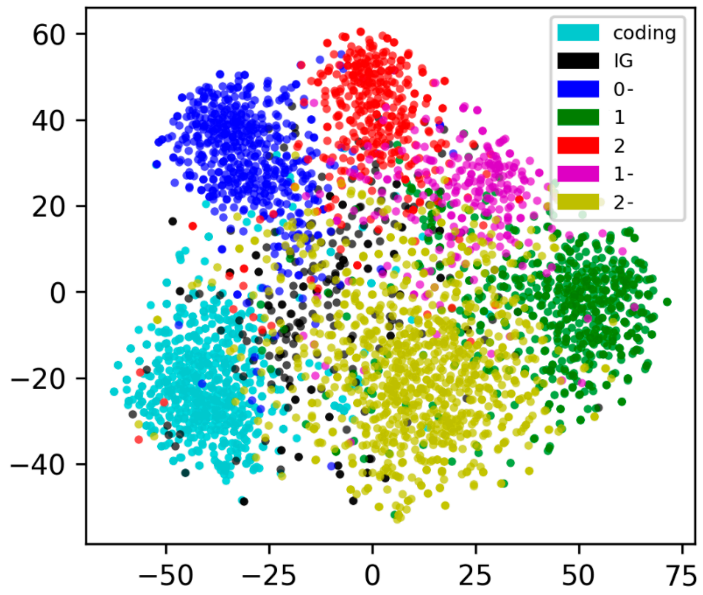

Supplementary Figure 2 A t-SNE plot of the EDPs of coding and noncoding ORFs for the representative genome Caulobacter phage. This is the same EDP data as in Figure 2B, but with the stop codon features excluded to better show the seven clusters. Each point is the EDP of an ORF in 20-dimensional space, and are colored according to whether they are coding, intergenic (IG), or for the noncoding: their relation to the coding frame (0−, 1, 2, 1−, 2−).

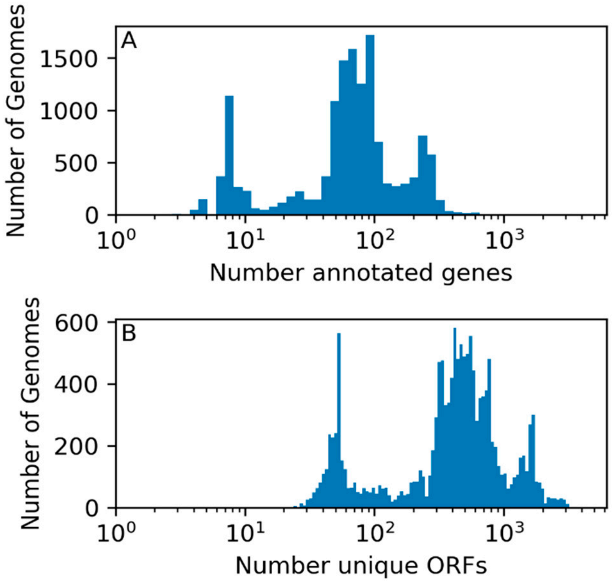

Supplementary Figure 3 Histograms showing the distribution of genome sizes in: **(A)** the number of genes in the consensus genome annotations **(B)** the number of potential unique ORFs, where a unique ORF is the single ORF represented by all possible start codon locations, which The number of annotated genes range from 2 to 1,044, and is analogus to *unique coding* ORFs, while *unique* ORF range from 18 to 4,720, and are *unique coding* ORFs (i.e. annotated genes) plus *unique noncoding* ORFs.

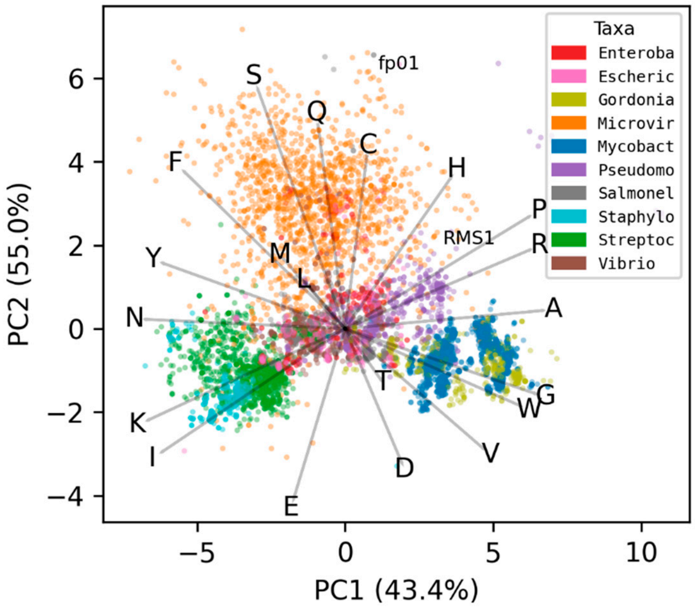

Supplementary Figure 4 The amino frequency of coding ORFs in phage genomes from the top 10 most abundant taxa, using the methodology as Figure 1B. As datasets often do not have full taxonomic lineages, the estimation was made according to the first 8 characters of the sequence name. This helps to merge cases like “Microviridae phage X” and “Microvirus phage Y” into the same category, but can potentially lead to over/under estimating taxa. Most taxa follow the linear horizontal trend corresponding to GC content, however the microviridae (and other taxa) deviate from the expected frequencies for the over observed amino acids (FSQC) and the under observed amino acids (EDV), suggesting that these taxa use an alternate codon table. The two examples of phages with unusual amino-acid compositions from Figure 3 are shown in the plot; where the Ralstonia phage RMS1 is slightly deviated from the expected trend, while the Escherichia phage fp01 is significantly diverged.
